# Direct Preparation of Cellulose Nanofibers from Bamboo by Nitric Acid and Hydrogen Peroxide Enables Fibrillation via a Cooperative Mechanism

**DOI:** 10.3390/nano10050943

**Published:** 2020-05-15

**Authors:** Jinlong Wang, Xusheng Li, Jianxiao Song, Kunze Wu, Yichun Xue, Yiting Wu, Shuangfei Wang

**Affiliations:** 1 Department of Light Industrial and Food Engineering, Guangxi University, Nanning 530004, China; long05360525@163.com (J.W.); zhuyingsongxing@163.com (J.S.); wkzli123@163.com (K.W.); hueiun@163.com (Y.X.); ww1031327514@163.com (Y.W.); wangsf@gxu.edu.cn (S.W.); 2 Guangxi Key Laboratory of Clean Pulp & Papermaking and Pollution Control, Nanning 530004, China

**Keywords:** cellulose nanofibers, biomass, nitric acid, hydrogen peroxide

## Abstract

Separating the fibers, deconstructing both the interlamellar structures and the intermicrofibrils structures in the cell wall, and cleaving the amorphous regions of cellulose (all reached in one bath chemical-assisted treatment), then extracting cellulose nanofibers (CNFs) from biomass, is both challenging and imperative. A simple, cost-effective and green strategy for extracting CNFs from bamboo using nitric acid and hydrogen peroxide (NCHP), to enable fibrillation via a cooperative mechanism, is demonstrated herein. NCHP-CNFs 13.1 ± 2.0 nm wide, with a high aspect ratio, 74% crystallinity, excellent UV resistance and high thermal stability, were successfully extracted by treatment in HNO_3_ aqueous solution, at a concentration of 3.2 mol/L, and treatment with 60.00 mmol/g H_2_O_2_ at 50 °C for 48 h. The yields of NCHP-CNFs reached 73% and 99% based on biomass and cellulose, respectively, due to the high delignification selectivity of OH^+^ and the mild aqueous conditions during the NCHP treatment. These NCHP-CNFs with excellent UV resistance can potentially be applied in the field of UV-resistant coatings, to replace organic and inorganic materials.

## 1. Introduction

Cellulose nanofibers (CNFs) have excellent properties, such as extremely large specific surface areas, high Young’s moduli, high specific strengths, low densities, and low thermal expansion coefficients, and thus the research and development of CNFs has been driven by both industry and academia [1]. Potential applications of CNFs include water purification [2,3], nanopaper [4], aerogels [5], gas barrier films [6], pharmaceuticals [7,8], and other high-performance and high-tech nanomaterials.

Cellulose is a natural linear macromolecule consisting of 300–15,000 D-glucose units, linked through β-1,4-glycoside bonds [9]. Due to their molecular weight and the intercellulose hydrogen bonds (three hydroxyl groups per glucose unit), cellulose chains easily aggregate into bundles. Generally, 30–40 cellulose chains are aggregated into completely separate primary microfibrils 3–4 nm wide with crystalline and amorphous regions. The microfibrils 10–20 nm wide are composed of a few to dozens of primary microfibrils. The microfibers are embedded in a matrix consisting of lignin and hemicellulose, and the cell walls are assembled by unique hierarchical structures through intermicrofibrils hydrogen bonds and cohesion to form firm structures in biomass [10]. CNFs are usually extracted from biomass by chemical-assisted mechanical treatment processes. Repeated mechanical treatments, such as homogenization, sonication and microfluidization, are often required to cleave interfibrillar hydrogen bonds to enable fibrillation, and these processes consume a large amount of energy making them prohibitively expensive for commercial applications [9].

Various chemical-assisted methods involving various plant materials have been extensively studied at the bench scale. The consensus on effective strategies for reducing the cost of CNF production focuses on the following. (1) Chemical-assisted treatments including acid hydrolysis with H_2_SO_4_ and cellulase pretreatment. These methods enable fibrillation by cleaving the cellulose chains in the amorphous regions. However, the yields based on cellulose of acid hydrolysis with H_2_SO_4_ are low at only 25–35% [11], and the operating conditions for cellulase pretreatment are harsh [12]. (2) Introducing charged functional groups on the surface of the fibers to form a strong electrostatic repulsion between microfibrils in water, which can reduce the energy needed for mechanical treatment. Traditionally significant and representative methods for introducing charged groups onto fibers are TEMPO oxidation [13,14], periodate-chlorite oxidation [15,16], carboxymethylation [17], and quaternization [18]. These methods are only effective for cellulose raw materials, so various pretreatment procedures are required to extract cellulose fibers from biomass. To remove lignin, hemicellulose, and other components from biomass, typical pretreatment procedures, such as solvent treatment, alkali pretreatment, and bleaching, have been reported. However, typical methods for converting biomass into CNFs comprise multistep chemical-assisted treatments requiring multiple reagents and repeated mechanical processes that sometimes require substantial amounts of water, electrical energy, and expensive or uncommon chemicals (such as 2,2,6,6-tetramethylpiperidine-1-oxyl radical) [19].

Hydrogen peroxide (H_2_O_2_) is a green and economic oxidant, which has also been applied in various fields [20]. In recent years, H_2_O_2_ has appeared as a chemical additive in the field of CNF preparation. H_2_O_2_ in combination with ferrous sulfate (FeSO_4_) to produce CNFs from cellulose fibers has been demonstrated by Li et al. [21] Wen et al. have reported that the ultraviolet light and H_2_O_2_ enhanced ozone (O_3_) treatment capacity to produce CNFs from cellulose fibers [22]. Importantly, the oxidation effectiveness of HNO_3_ could be enhanced by the addition of H_2_O_2_, due to the fact that hydrogen peroxide can oxidize HNO_2_ to HNO_3_ [23].

In this work, a simple method to extract CNFs from bamboo in one bath with nitric acid and hydrogen peroxide (NCHP) under mild aqueous conditions was demonstrated. The NCHP process can effectively separate the fibers from bamboo and progressively deconstruct the interlamellar layers and the intermicrofibrils in the cell wall by removing the lignin and hemicellulose. During the NCHP treatment, the fiber width decreased from 450 μm to 10 μm, and the crystallinity of cellulose increased from 54% to 74%. The homogenization process proceeded smoothly without clogging. The NCHP-CNFs extracted from bamboo by the NCHP process are 13.1 ± 2.0 nm wide with high aspect ratios, 74% crystallinity, and high thermal stability, as well as the excellent ability of the obtained NCHP-CNFs to absorb the UV spectrum. Due to the high delignification selectivity of OH^+^ and the mild aqueous conditions during the NCHP treatment, the yields of NCHP-CNFs can reach 73% and 99%, based on biomass and cellulose, respectively. The NCHP process is conducted in one bath with common chemicals instead of a multistep, multichemical processes, greatly simplifying the preparation steps and reducing the consumption of chemicals, energy, and water. The residual liquid from the NCHP process could be effectively neutralized with base to produce nitrate fertilizers [19]. The available raw materials for NCHP-CNFs include a vast amount of waste biomass, such as shrubs and agricultural wastes, which may lead to lower costs. Thus, the NCHP process is a simple, cost-effective and green pathway to extract CNFs from biomass. The applicability of NCHP-CNFs is based on their excellent UV resistance, making them suitable for UV-resistant coatings to replace organic and inorganic materials.

## 2. Materials and Methods

### 2.1. Chemicals and Raw Materials

A three-year-old bamboo plant (Bambusa chungii) provided by the planting base of Guangxi Zhuang Autonomous Region Forestry Science Research Institute was used as the raw material (cellulose, lignin, and hemicellulose contents: 48.05%, 22.09%, and 28.88%, respectively). The bamboo was crushed to pass through 40–60 mesh, and the material was air-dried and then stored in a sealed bag. The chemicals used in the experiment, i.e., nitric acid (HNO_3_, 65 wt. %) and hydrogen peroxide (H_2_O_2_, 30 wt. %), were all analytically pure reagents purchased from Nanning Blue Sky Experimental Equipment Co., Ltd. (Naning, China)

### 2.2. Preparation of the Cellulose Nanofibers 

To prepare the NCHP-CNFs, 5 g of powdered bamboo and a 60 mmol/g dosage of H_2_O_2_ base on bamboo mass were added to 200 mL of 3.2 mol/L or 9.6 mol/L HNO_3_ with magnetic stirring. The reaction was carried out at 50 °C (35 or 65 °C) for 48 h or 72 h and was terminated by adding deionized water (five times the volume of the reaction). To obtain the cellulose fibers, the reaction solution was washed with deionized water under suction, until the filtrate was neutral. The resulting cellulose fibers were then diluted to 0.8% and homogenized using a high-pressure homogenization chamber with a pore diameter of 87 μm and a homogenization pressure of 1500 bar. This operation was repeated 5 times to obtain the NCHP-CNF sample.

### 2.3. Characterization

The chemical compositions of the bamboo powder and NCHP fibers were determined according to the method described by the Technical Association of the Pulp & Paper Industry Inc. (Atlanta, GA, USA) The cellulose content was measured using TAPPI standard T203 OS-74, and the lignin content was measured using TAPPI standard T222 OS-83.

FTIR (Fourier-transform infrared spectroscopy) spectra were recorded using an FTIR spectrometer (TENSOR II, Brook Technology, Ettlingen, Germany) over a wavenumber range from 4000 to 400 cm^−1^, with a resolution of 4 cm^−1^.

SEM (scanning electron microscopy) was conducted using an SU8220 instrument (Hitachi, Tokyo, Japan), and the sample was sputter-coated with gold before observation.

TEM (transmission electron microscopy) was performed on a JEM-1200EX instrument (JEOL, Tokyo, Japan), at an acceleration voltage of 200 kV. The NCHP-CNFs solution was diluted to a concentration of 0.008% and 1 μL of the solution was dropped on a copper mesh to be naturally dried. Then, phosphotungstic acid was added dropwise for 20 min, and the excess dye was removed by filter paper. The diameter of the NCHP-CNFs was measured at least 100 times in the TEM image using Nano Measurer software (Version 1.2.5, San Francisco, CA, USA).

The lyophilized sample needs to be milled into powder using an agate mortar. The crystallinities of the raw materials and samples were analyzed using a benchtop XRD (X-ray diffractometer) (MINFLEX 600, Tokyo, Japan). The untreated powdered bamboo was evaluated by powder X-ray diffraction, while the NCHP fibers were evaluated as films. The crystallinity index (CrI) for each sample was calculated from the XRD patterns, according to the conventional method [24,25].

The lyophilized sample needs to be milled into powder using an agate mortar. 10 mg powder was added to the pan for TGA. TGA (thermogravimetric analysis) was carried out at a heating rate of 10 °C min^−1^ under a nitrogen atmosphere in a synchronous thermal analyzer (NETZSCH STA 449F5, Selb, Germany), over a temperature range of 30–600 °C. The sample was dried at 105 °C for 3 h to remove moisture before the test. 

## 3. Results and Discussion

### 3.1. Proposed Mechanism of Pretreatment 

**Figure 1:**
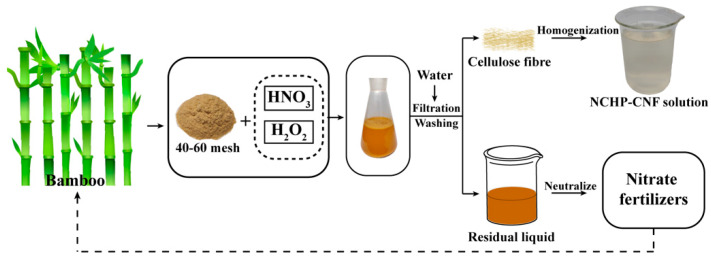
Schematic diagram of the extraction of cellulose nanofibers (CNF) from bamboo, by nitric acid and hydrogen peroxide (NCHP) treatment.

A schematic diagram of the CNFs extracted from bamboo by the NCHP treatment is shown in Figure 1.

**Figure 2:**
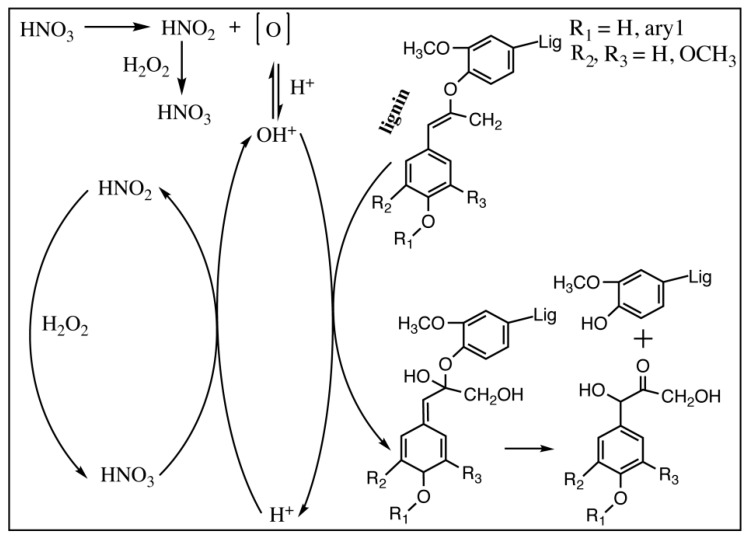
Proposed mechanism of pretreatment.

HNO_3_ is a strongly oxidizing inorganic acid and tends to ionize, releasing H^+^, in aqueous solutions. Due to the structural asymmetry of HNO_3_, the N–O bond is weak, and it can be readily cleaved to release atomic oxygen [O], by the anti-polarization of H^+^ [26]. Atomic oxygen [O] combines with H^+^ to form OH^+^. OH^+^ can react as an electrophile. Due to the decomposition of HNO_3_, the HNO_2_, NO_2_ and NO were generated, and the decomposition process could be accelerated by temperature, which changed the solution to yellow and released brown NO_2_ gas [27]. Here, no brown gas (NO_2_) was observed in the NCHP treatment (Figure S1), but it was in HNO_3_ and NaNO_2_ treatment [19]. This indicates that the H_2_O_2_ inhibits the formation of NO_2_. This may be explained as being because H_2_O_2_ acts as the active oxidant and it can oxidize HNO_2_ to HNO_3_ [28,29]. HNO_3_, as an active oxidant, was reduced to HNO_2_ during the NCHP treatment, while H_2_O_2_ regenerated HNO_3_ from HNO_2_ and it was consumed as the reaction continued. A diagram of the proposed delignification mechanism for NCHP treating is shown in Figure 2.

The lignin macromolecules, which have a high content of electron-rich functional groups, including olefins, carbonyls, and aromatic ring structures, were attacked by OH^+^. These electrophilic reactions caused the lignin to fragment into small molecules that dissolved from biomass, allowing the lignin to be removed from the biomass [30]. The ether bonds of hemicellulose are easy to break and lead to hemicellulose degradation under acidic conditions [31]. Amorphous cellulose was degraded by acid hydrolysis and oxidative fracture in the NCHP solution. Cellulose is generally stable in the NCHP solution, due to the protection of the crystalline cellulose regions and the mild aqueous conditions. The exact mechanism of preparing the NCHP-CNFs from biomass was investigated further.

### 3.2. Chemical Composition Analysis 

Several variables, including temperature, time, H_2_O_2_ dosage and HNO_3_ concentration, were investigated by the analysis of the yields, the contents and retention ratios of cellulose, and the contents and removal ratios of lignin and hemicellulose. The experimental data are presented in Table 1.

As seen from Table 1, the removal ratios of lignin in the HNO_3_ aqueous solutions at concentrations of 3.2 and 9.6 mol/L were 74.14% (S.N.2) and 99.01% (S.N.1), respectively. As the time of NCHP treatment was prolonged, the lignin removal ratios increased from 74.14% for 48 h (S.N.2) to 85.45% for 72 h (S.N.3). As the temperature of treatment was increased, the ratios of lignin removal increased from 65.48% at 35 °C (S.N.6) to 74.14% at 50 °C (S.N.2) and then to 84.43% at 65 °C (S.N.5). The percentages of lignin removal were 74.14% (S.N.2) and 78.8% (S.N.4) by NCHP treatment with 60 and 90 mmol/g H_2_O_2_, respectively. This phenomenon indicates that the HNO_3_ concentration, the time, the temperature and H_2_O_2_ promote lignin removal. The hemicellulose removal percentages under all but the 35 °C conditions were in the range of 73.99–86.4%. The experimental results show that lignin and hemicellulose could be effectively removed from bamboo by NCHP treatment.

As seen from Table 1, the cellulose retention ratios were 96.96% (S.N.2) and 39.56% (S.N.1) when the HNO_3_ concentrations were 3.2 and 9.6 mol/L, respectively. As the time of NCHP treatment was prolonged, the cellulose retention ratios decreased from 96.96% for 48 h (S.N.2) to 91.62% for 72 h (S.N.3). As the temperature of treatment was increased, the ratios of cellulose retention decreased from 99.85% at 35 °C (S.N.6) to 96.96% at 50 °C (S.N.2) and then to 87.11% at 65 °C (S.N.5). The percentages of cellulose retention were 96.96% (S.N.2) and 95.45% (S.N.4) by NCHP treatment with 60 and 90 mmol/g H_2_O_2_, respectively. These results indicate that increasing the HNO_3_ concentration, temperature, time and H_2_O_2_ dosage all accelerate the degradation of cellulose. The experimental results showed that the NCHP process has a high cellulose retention ratio.

It can be seen from S.N.6 that the yield of fibers can reach 73.44%, which was attributed to the retentions of cellulose (99.85%), hemicellulose (65.41%) and lignin (34.52%), under mild aqueous conditions. Considering the chemical consumption, the removal of hemicellulose and lignin, the cellulose retention, etc., NCHP-CNFs were prepared by treatment with aqueous HNO_3_, at a concentration of 3.2 mol/L and a dosage of 60.00 mmol/g H_2_O_2_ at 50 ℃ for 48 h.

### 3.3. Fiber Morphological Characterization 

**Figure 3:**
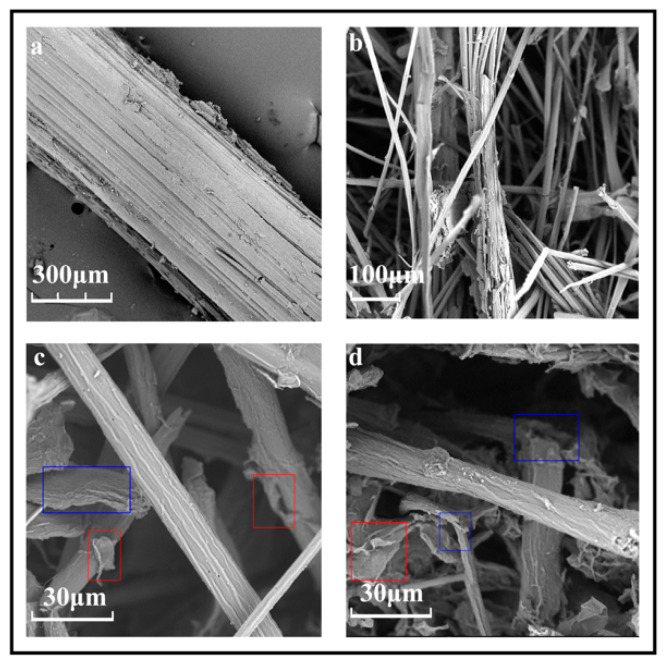
SEM image of the morphology of the fibers after NCHP treatment for different times: (**a**) bamboo powder; (**b**) 12 h; (**c**) 24 h; and (**d**) 48 h.

The morphologies of the bamboo powder and fibers after NCHP pretreatment at 50 °C for 12, 24, and 48 h observed by SEM were compared, as shown in Figure 3. Bamboo powder with a particle size of approximately 450 μm was selected by 40–60 mesh, and it had a compact surface structure, as shown in Figure 3a. It can be seen from Figure 3b that a large portion of the fibers that had been separated were approximately 10 μm wide after NCHP treatment for 12 h; the fiber bundles that had not yet been fully separated were less than 50 μm and had become loose. This was attributed to the lignin and hemicellulose in the intercellular layer being removed during the NCHP treatment. It can be seen from Figure 3c that the fibers had been completely separated; some of the fibers had atrophied (highlighted in blue), and the lamellar fibers detached from the cell wall (highlighted in red) had been obtained, but the overall morphology of the fibers remained intact. This indicated that the structures of the interlamellar and intermicrofibrils in the cell wall had been progressively destroyed, and this was attributed to the removal of lignin and hemicellulose, which had penetrated deep into the cell wall. Figure 3d shows that more lamellar fibers were present (highlighted in red) and fiber kinks and deformities (highlighted in blue) were observed after NCHP treatment for 48 h. This indicated that the hierarchical structure of the cell wall was further degraded, and the structure of the microfibers had been partially destroyed, due to the amorphous regions of the cellulose chains breaking. The reduction of the microfibril size and the cleavage of the cellulose chains in the amorphous regions were conducive to subsequent nanofibril formation by high-pressure homogenization. In fact, the homogenization process proceeded smoothly without clogging.

The energy consumption of CNF extraction from biomass by the NCHP and TEMPO processes at the bench scale was measured as shown in Figure S2. Figure S2 shows that the energy required to produce CNFs by NCHP (0.69 kWh/g) was far lower than that of the TEMPO process (14.49 kWh/g) at the bench scale [19]. This was attributed to the fact that the NCHP method used one bath and common chemicals while the TEMPO process requires multiple steps and multiple uncommon chemicals, resulting in a higher consumption of both chemicals and energy. Furthermore, the wastewater could be utilized as a nitrogen fertilizer following neutralization with a base [19]. The available raw materials for NCHP-CNFs include a variety of waste biomass resources, such as shrubs and agricultural wastes, which may lead to lower costs. 

### 3.4. Chemical and Morphological Characterization 

**Figure 4:**
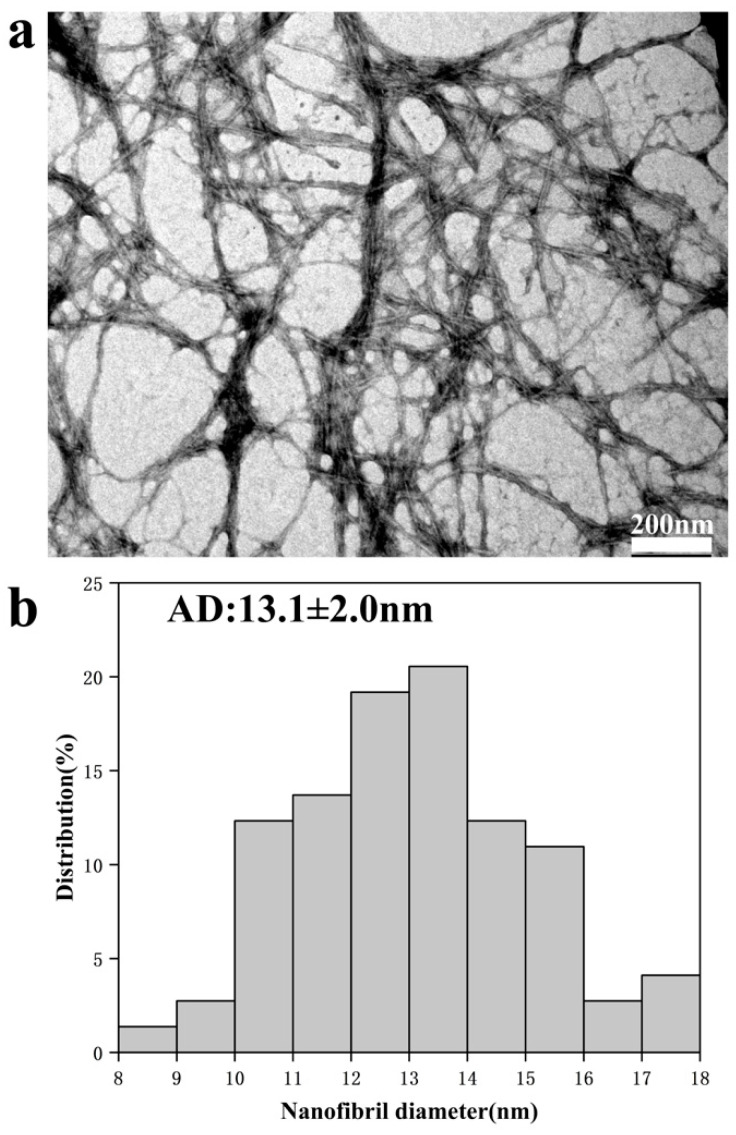
(**a**) TEM image of the NCHP-CNFs; (**b**) diameter distribution of the NCHP-CNFs.

The morphology of the NCHP fibers after high-pressure homogenization observed by TEM and their diameters as measured by Nano Measurer software are shown in Figure 4. It can be seen from Figure 4 that the NCHP-CNFs exhibited high aspect ratios, a certain amount of flocculation, superposition, and a network structure due to the fibrillation process, and the free hydroxyl groups on the surfaces promoted the aggregation of the NCHP-CNFs [32,33]. The NCHP-CNFs were similar to TEMPO-CNFs, with respect to their high aspect ratios, flocculation, superposition, and network structure [13]. Figure 4 shows that the fibers had cross section diameter of 13.1 ± 2.0 nm. This indicates that the NCHP-CNFs were prepared successfully.

**Figure 5:**
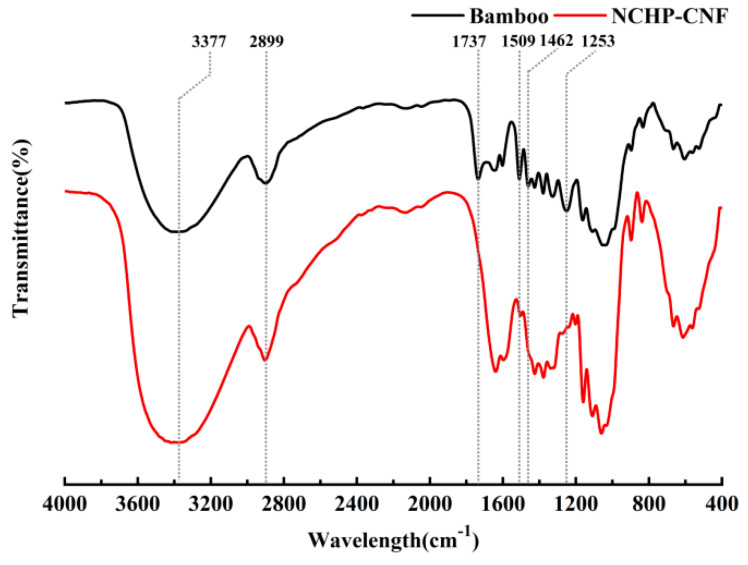
FTIR spectra of the untreated bamboo powder fiber and the NCHP-CNFs.

The FTIR spectra of the bamboo powder and the NCHP-CNFs were compared, as shown in Figure 5. Figure 5 shows that the characteristic absorption peaks of cellulose at 3377 cm^−1^ (OH stretching), 1642 cm^−1^ (OH bending) and 2899 cm^−1^ (C–H stretching) were significantly strengthened from the bamboo powder to the NCHP-CNFs, due to the increase in the cellulose content. This indicates that the chemical structure of cellulose remains unchanged during NCHP treatment [34]. It can be seen from Figure 5 that the characteristic absorption peaks of lignin at 1509 cm^−1^ (basic structural framework of aromatic rings), 1462 cm^−1^ (C–H deformation), and 1253 cm^−1^ (aryl C–O stretching) were still observed [35], but their intensities were lower, due to the decrease in the lignin content (from 22% to 10%, Table 1). This indicates that the basic structural framework of the lignin matrix still existed after NCHP treatment. As shown in Figure 5, the characteristic absorption peak at 1737 cm^−1^ (C=O stretches for the acetyl and sugar ester groups in hemicellulose, the ferulic acid, and the ester carboxyl group) was present in the spectrum of bamboo, but was not present in the spectrum of NCHP-CNFs [36,37]. This can be explained by the fact that hemicellulose, lignin, and a few other components containing C=O groups were dissolved during NCHP treatment.

**Figure 6:**
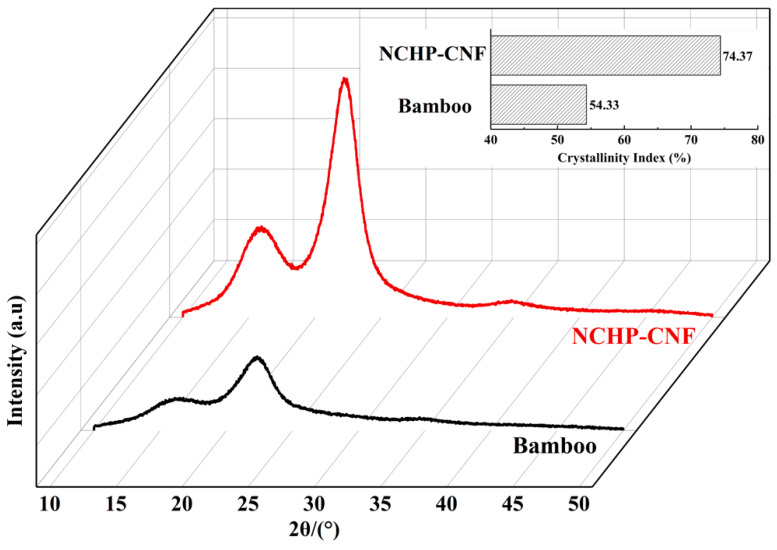
XRD patterns of the bamboo powder fiber and the NCHP-CNFs.

The crystallinity and crystal structure of cellulose usually change during chemical pretreatment and mechanical processing [38,39]. The CrIs calculated by the Segal equation and the XRD patterns of NCHP fibers and bamboo powder are compared in Figure 6. The diffraction peaks at 16° (101), 22° (002), and 34° (004) for cellulose I were significantly strengthened after NCHP treatment, as shown in Figure 6. This indicates that the crystal structure of the fibers was unchanged by NCHP treatment [24]. As seen from Figure 6, the CrIs of the bamboo powder and NCHP-CNFs were 54.33% and 74.37%, respectively. This indicates that the proportion of amorphous to crystalline regions in the cellulose was reduced during NCHP treatment. This was attributed to the fact that amorphous lignin and hemicellulose, as well as the amorphous regions of cellulose, were removed during NCHP treatment [40]. A high crystallinity corresponds to the improved strength and thermal stability of the cellulose nanofibers [41,42].

**Figure 7:**
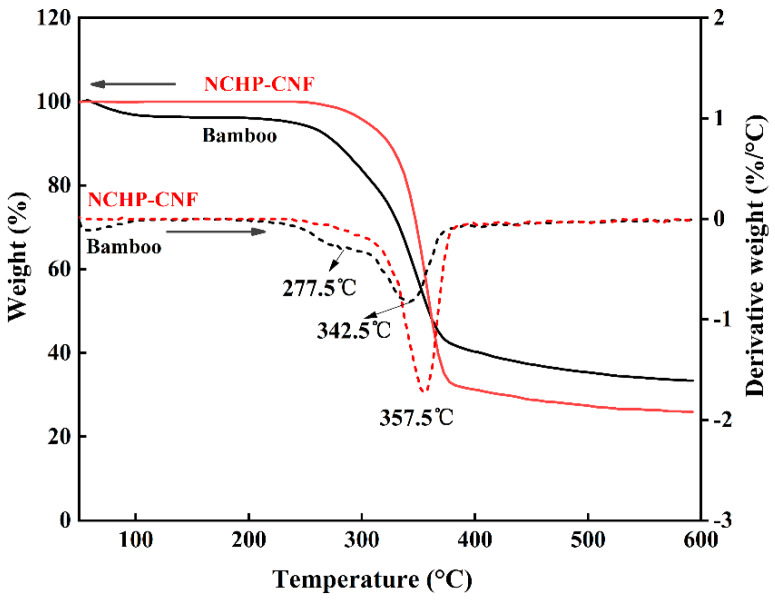
TGA curve and derivative curve of the bamboo powder fiber and the NCHP-CNFs.

To investigate the thermal stability of the NCHP-CNFs obtained in this study, a thermogravimetric analysis was performed. The TGA record and derivative curve of the bamboo powder fiber and NCHP-CNF are shown in Figure 7. At 100 °C on the TG curve, the slight weight loss of the powdered bamboo fiber is attributed to the evaporation of moisture in the sample [43]. The TG curve shows that the decomposition of bamboo powder was earlier than NCHP-CNFs, and the residual material of bamboo powder was higher than NCHP-CNFs over 600 °C. The two phenomena are attributed to pyrolysis of hemicellulose and high lignin content, respectively [44]. The differential TG curve more accurately shows the difference in pyrolysis between the powdered bamboo fibers and NCHP-CNFs. Due to the pyrolysis of hemicellulose, the powdered bamboo fibers have distinct low temperature shoulders, which correspond to the mass loss on the TG curve at low temperatures. The results show that lignin and hemicellulose could be effectively removed from bamboo by NCHP treatment. The main peaks of the bamboo powder and NCHP-CNFs are 342 °C and 357 °C, respectively, representing the pyrolysis of cellulose. In general, the thermal stability of cellulose will decrease with the decrease of the size of cellulose particles. The good thermal stability of the NCHP-CNFs is attributed to the high crystallinity and the presence of lignin [19,45,46]. The good thermal stability of the NCHP-CNFs make them more broadly applicable [46].

In addition, the UV transmittance of NCHP-CNF paper was 0%, as shown in Figure S3. This was attributed to the fact that the lignin remaining in the CNFs is rich in conjugated systems, enabling excellent UV resistance [46,47]. This result suggests that the NCHP-CNFs will be applicable in the field of UV-resistant coatings, to replace organic and inorganic materials.

## 4. Conclusions

Separating the fibers, deconstructing both the interlamellar structures and the intermicrofibrils structures in the cell wall, and cleaving the amorphous regions of cellulose (all reached in one bath chemical-assisted treatment), then extracting cellulose nanofibers (CNFs) from biomass, is both challenging and imperative. In this work, the effects of several variables, including temperature, time, H_2_O_2_ dosage and HNO_3_ concentration, were investigated by analyzing changes in the chemical composition of the obtained fibers. SEM, TEM, XRD, FTIR and TGA were used to characterize the NCHP-CNFs. The results are as follows. The practicality of the NCHP process for extracting CNFs directly from bamboo in one bath was demonstrated. CNFs 13.1 ± 2.0 nm wide, with a high aspect ratio, 74% crystallinity, and high thermal stability were successfully obtained via treatment with 3.2 mol/L aqueous HNO_3_ and 60.00 mmol/g H_2_O_2_ at 50 °C for 48 h. The yields of NCHP-CNFs can reach 73% and 99% based on biomass and cellulose, respectively. The NCHP process greatly simplified the preparation of CNFs. The application potential of the NCHP-CNFs is based on their excellent ability of UV resistance, which makes them suitable replacements for organic and inorganic materials in the field of UV-resistant coatings.

## Figures and Tables

**Table 1:** The conditions of bamboo chemical pre-treatment with NCHP as well as the yield, chemical component ratio, removal ratio.

S.N.	Treatment Condition (h/°C)	Nitric Acid Concentration (mol/L)	Hydrogen Peroxide (mmol/g)	Yield wt (%)	Lignin Content (%)	Lignin Removal (%)	Cellulose Content (%)	Cellulose Retention (%)	Hemicellulose Content (%)	Hemicellulose Removal (%)
1	48/50	9.6	60.00	23.47	0.37	99.01	80.99	39.56	18.64	84.85
2	48/50	3.2	60.00	58.72	9.65	74.14	79.34	96.96	11.01	77.61
3	72/50	3.2	60.00	53.13	5.43	85.45	82.86	91.62	11.71	78.46
4	48/50	3.2	90.00	56.01	7.91	78.80	78.68	95.45	13.41	73.99
5	48/65	3.2	60.00	48.61	5.81	84.43	86.11	87.11	8.08	86.40
6	48/35	3.2	60.00	73.44	12.88	65.48	65.33	99.85	21.79	44.59

## Supplementary Materials

The following are available online at https://www.mdpi.com/2079-4991/10/5/943/s1, Figure S1: Bamboo powder reaction process solution, Figure S2: Process flowchart for estimating the energy consumption and chemical consumption of preparing NCHP-CNFs based on the nitric acid-hydrogen peroxide method according to laboratory conditions, Figure S3: UV-Vis transmittance of the NCHP-CNFs.
