# Supplementary material for: Direct Preparation of Cellulose Nanofibers from Bamboo by Nitric Acid and Hydrogen Peroxide Enables Fibrillation via a Cooperative Mechanism
Source: Nanomaterials (Basel). 2020 May 15;10(5):943. doi: 10.3390/nano10050943 (PMC7279225; doi:10.3390/nano10050943)
Supplement: Supplementary file 1 [file nanomaterials-10-00943-s001.pdf]

Supplementary information

Direct Preparation of Cellulose Nanofibers from Bamboo by Nitric Acid and Hydrogen Peroxide Enables Fibrillation via a Cooperative Mechanism

Jinlong Wang [1,2], Xusheng Li [1,2,*], Jianxiao Song [1,2], Kunze Wu [1,2], Yichun Xue [1,2], Yiting Wu [1,2] and Shuangfei Wang [1,2]

[1] Department of Light Industrial and Food Engineering, Guangxi University, Nanning 530004, China; long05360525@163.com (J.W.); zhuyingsongxing@163.com (J.S.); wkzli123@163.com (K.W.); hueiun@163.com (Y.X.); ww1031327514@163.com (Y.W.); wangsf@gxu.edu.cn (S.W.)

[2] Guangxi Key Laboratory of Clean Pulp & Papermaking and Pollution Control, Nanning 530004, China

* Correspondence: lixusheng@gxu.edu.cn; Tel.: +86-0771-3237301

Received: 18 April 2020; Accepted: 13 May 2020; Published: date

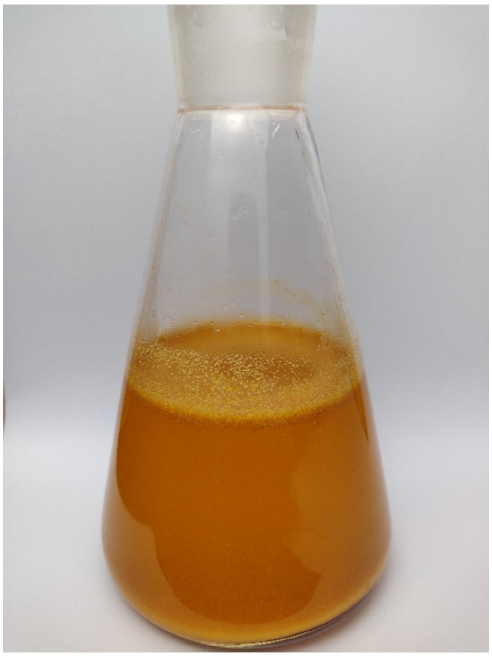

Figure 1. Photo of the reaction process of bamboo powder treated by NCHP.

The photo of the reaction process of bamboo powder was treated in HNO_3 aqueous solution at a concentration of 3.2 mol/L and with a dosage of 60.00 mmol/g H_2O_2 at 50 °C for 3 h is shown in Figure S1. As seen from Figure S1, there no brown gas clearly observed in the gas above the Erlenmeyer flask.

The NCHP-CNF were prepared under laboratory conditions using untreated powdered bamboo. The NCHP-CNF (at 48 h) were used as examples to simply estimate the cost of electricity and chemicals in the production of the nanofibers. These estimated costs will be greatly reduced for large-scale production, demonstrating the great potential of the NCHP process in industrialization. In the experiment, we did not include calculations of the cost of equipment and labor. The instruments involved in the experiment were very simple, and there are no obstacles in achieving scaled production. The laboratory instruments used in this study were: Collector constant temperature magnetic agitator (Shanghai Yuhua DS-101S, Shanghai, China): 220 V, 50 Hz; Circulating water vacuum pump (Zhengzhou Greatwall, Zhengzhou, China): 220 V, 1.3 A, and 50 Hz; Homogenizer (Microfluidics): 220 V, 50 Hz.

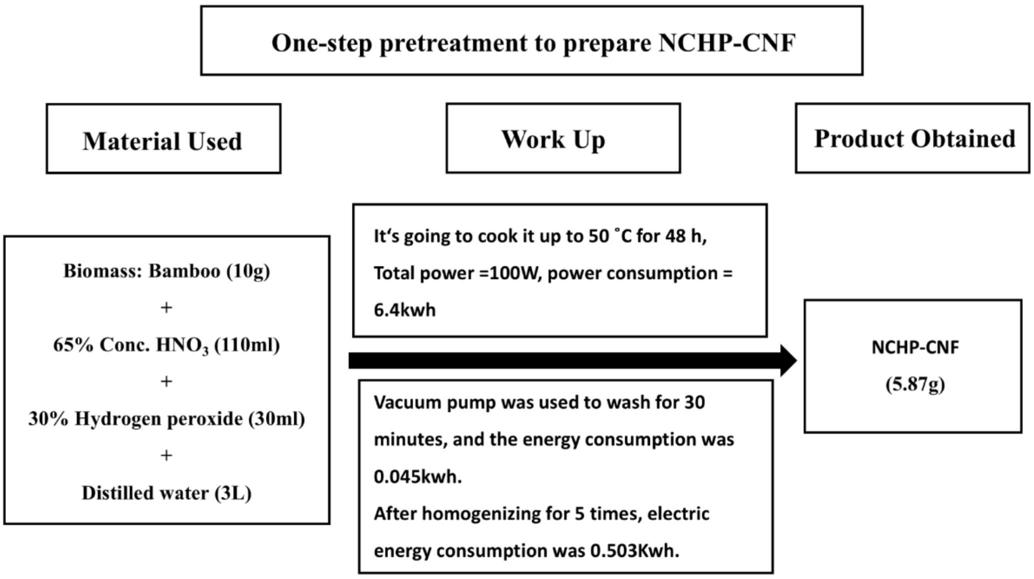

Figure 2. Process flowchart for estimating the energy consumption and chemical consumption of preparing NCHP-CNFs based on the nitric acid-hydrogen peroxide method according to laboratory conditions.

Figure S2 presents a processcording to Sharma [1] , the study of 1 g biomass by the TEMPO-oxidation method to obtain NCNF consumes 14.49 kWh; the energy required by the proposed NCHP method is much lower than that of the TEMPO-oxidation method, according to calculation, the production of 1 g of NCHP-CNFs consumes 1.18 kWh. Furthermore, the amount of chemicals used is greatly reduced compared to conventional processes.

The suspension containing 0.24 g of absolute dry weight CNF is then diluted to a concentration of 0.1% and stirred until the fibers are completely dispersed. A polytetrafluoroethylene film, with a pore size of 0.22 μm, was placed in a G5 core funnel and vacuum-filtered to form a film. The peeled wet film was sandwiched between two pieces of filter paper and the stack was pressed between the two glass plates under a certain pressure for 24 h. Next, the film was moved to a drying oven at 60 °C for 8 h. The UV absorption and transmittance of the samples were measured with a UV-Vis spectrophotometer (U-4100, Hitachi High-Tech, Tokyo, Japan) with an integrating sphere. UV-Vis transmittance of the NCHP-CNFs film was shown in Figure S3.

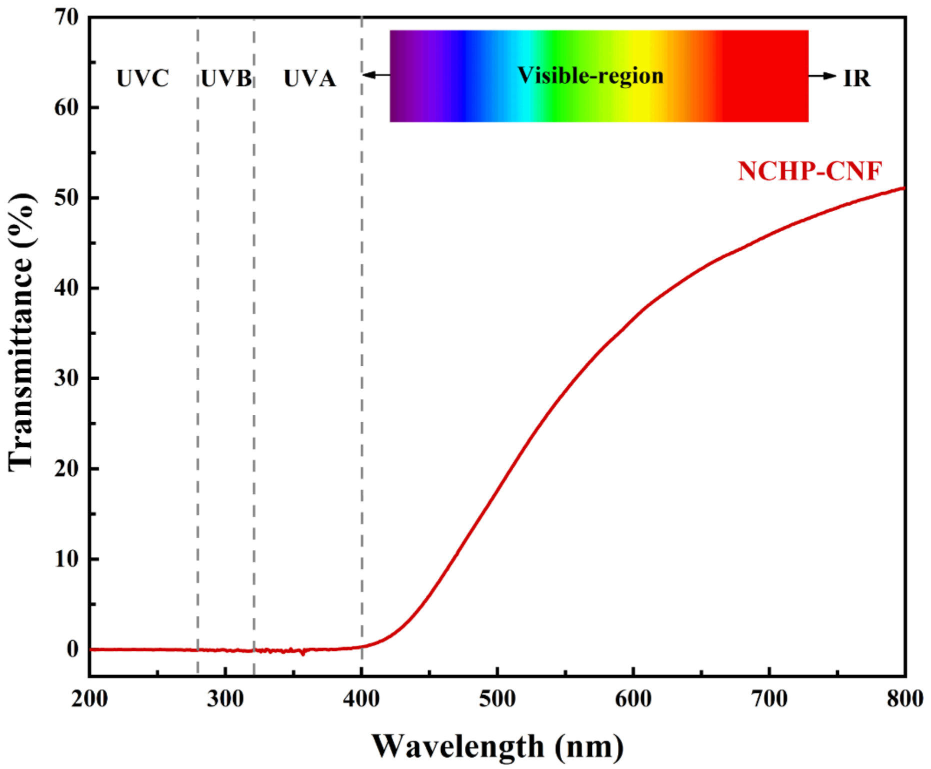

Figure 3. UV-Vis transmittance of the NCHP-CNFs.

Lignin, as a natural antiultraviolet material, is rich in phenolic structures and has excellent ultraviolet absorption capacity. The ultraviolet solar spectrum is traditionally divided into three bands: ultraviolet-A (315–400 nm), ultraviolet-B (280–315 nm), and ultraviolet-C (200–280 nm) [2]. As seen from Figure S3, The NCHP-CNFs film completely blocked all UV-A, UV-B and UV-C. This indicates that the excellent ability of the obtained NCHP-CNFs to absorb the UV spectrum.

References

1. Sharma, P.R.; Joshi, R.; Sharma, S.K.; Hsiao, B.S. A Simple Approach to Prepare Carboxycellulose Nanofibers from Untreated Biomass. *Biomacromolecules* **2017**, *18*, 2333–2342, doi:10.1021/acs.biomac.7b00544.
2. Paul, N.D.; Gwynn-Jones, D. Ecological roles of solar UV radiation: towards an integrated approach. *Trends Ecol. Evol.* **2003**, *18*, 48–55.
